# Clinical relevance of single item quality of life indicators in cancer clinical trials

**DOI:** 10.1054/bjoc.2001.1785

**Published:** 2001-05

**Authors:** J Bernhard, M Sullivan, C Hürny, A S Coates, C-M Rudenstam

**Affiliations:** IBCSG Coordinating Center, Effingerstr. 40, CH-3008 Bern, Switzerland; Health Care Research Unit, Institute of Internal Medicine, Sahlgrenska University Hospital, SE 413 45 Göteborg, Sweden; Geriatrics, Bürgerspital, Rorschacher Strasse 94, 9000 St. Gallen, Switzerland; Australian Cancer Society and University of Sydney, GPO Box 4708, Sydney NSW 2001, Australia; Department of Surgery, Sahlgrenska University Hospital, SE 413 45 Göteborg, Sweden

**Keywords:** breast cancer, quality of life, scale comparison, responsiveness, global indicators, randomized controlled trials

## Abstract

We investigated the hypothesis that global single-item quality-of-life indicators are less precise for specific treatment effects (discriminant validity) than multi-item scales but similarly efficient for overall treatment comparisons and changes over time (responsiveness) because they reflect the summation of the individual meaning and importance of various factors. Linear analogue self-assessment (LASA) indicators for physical well-being, mood and coping were compared with the Hospital Anxiety and Depression Scale (HAD), the Mood Adjective Check List (MACL) and the emotional behaviour and social interaction scales of the Sickness Impact Profile (SIP) in 84 patients with early breast cancer receiving adjuvant therapy. Discriminant validity was investigated by multitrait-multimethod correlation, responsiveness by standardized response mean (SRM). Discriminant validity of the indicators was present at baseline but less under treatment. Responsiveness was demonstrated by the expected pattern among treatments (*P* = 0.008). In patients without chemotherapy, the SRMs indicated moderate (0.5–0.8) to large (>0.8) improvements in physical well-being (0.70), coping (0.92), HAD anxiety (0.89) and depression (1.19), and MACL mental well-being (0.68). In patients with chemotherapy for the first 3 months, small but clinically significant improvements (>).2) included mood (0.38), coping (0.41), HAD axiety (0.31) and MACL mental well-being (0.35). Patients with 6 months chemotherapy showed no changes. The indicators also reflected mood disorders (HAD) and marked psychosocial dysfunction (SIP) at baseline and under treatment according to pre-defined cut-off levels. Global indicators were confirmed to be efficient for evaluating treatments overall and changes over time. The lower reliability of single as opposed to multi-item scales affects primarily their discriminant validity. This is less decisive in large sample sizes. © 2001 Cancer Research Campaign http://www.bjcancer.com


*British Journal of Cancer* (2001) **84**(9), 1156–1165
© 2001 Cancer Research Campaign
doi: 10.1054/ bjoc.2001.1785, available online at http://www.idealibrary.com on IDEAL®

### Clinical relevance of single item quality of life indicators in cancer clinical trials

**J Bernhard[1], M Sullivan[2], C Hürny[3], AS Coates[4] and C-M Rudenstam[5]**

[1]IBCSG Coordinating Center, Effingerstr. 40, CH-3008 Bern, Switzerland; [2]Health Care Research Unit, Institute of Internal Medicine, Sahlgrenska University Hospital, SE 413 45 Göteborg, Sweden; [3]Geriatrics, Bürgerspital, Rorschacher Strasse 94, 9000 St. Gallen, Switzerland; [4]Australian Cancer Society and University of Sydney, GPO Box 4708, Sydney NSW 2001, Australia; [5]Department of Surgery, Sahlgrenska University Hospital, SE 413 45 Göteborg, Sweden

**Summary** We investigated the hypothesis that global single-item quality-of-life indicators are less precise for specific treatment effects (discriminant validity) than multi-item scales but similarly efficient for overall treatment comparisons and changes over time (responsiveness) because they reflect the summation of the individual meaning and importance of various factors. Linear analogue self-assessment (LASA) indicators for physical well-being, mood and coping were compared with the Hospital Anxiety and Depression Scale (HAD), the Mood Adjective Check List (MACL) and the emotional behaviour and social interaction scales of the Sickness Impact Profile (SIP) in 84 patients with early breast cancer receiving adjuvant therapy. Discriminant validity was investigated by multitrait-multimethod correlation, responsiveness by standardized response mean (SRM). Discriminant validity of the indicators was present at baseline but less under treatment. Responsiveness was demonstrated by the expected pattern among treatments ($P = 0.008$). In patients without chemotherapy, the SRMs indicated moderate (0.5–0.8) to large (>0.8) improvements in physical well-being (0.70), coping (0.92), HAD anxiety (0.89) and depression (1.19), and MACL mental well-being (0.68). In patients with chemotherapy for the first 3 months, small but clinically significant improvements (>).2) included mood (0.38), coping (0.41), HAD axiety (0.31) and MACL mental well-being (0.35). Patients with 6 months chemotherapy showed no changes. The indicators also reflected mood disorders (HAD) and marked psychosocial dysfunction (SIP) at baseline and under treatment according to pre-defined cut-off levels. Global indicators were confirmed to be efficient for evaluating treatments overall and changes over time. The lower reliability of single as opposed to multi-item scales affects primarily their discriminant validity. This is less decisive in large sample sizes. © 2001 Cancer Research Campaign http://www.bjcancer.com

**Keywords**: breast cancer; quality of life; scale comparison; responsiveness; global indicators; randomized controlled trials

In implementing quality-of-life (QL) endpoints in cancer clinical trials, the plea for practical measures has become commonplace. The Australian New Zealand Breast Cancer Trials Group (ANZBCTG) and the International Breast Cancer Study Group (IBCSG) use a limited set of patient-rated indicators for assessing the impact of chemo- and endocrine therapy on QL in breast cancer clinical trials. These are single-item measures in the linear analogue self-assessment (LASA) format (Priestman and Baum, 1976), also known in social sciences as visual analogue scales (VAS).

The advantages of simple LASA indicators for data collection are clear-cut. However, these measures are generally expected to have lower reliability (i.e., less statistical precision) than sound multi-item measures (McHorney et al, 1992), resulting in lower responsiveness. For example, in an extensive investigation of a LASA indicator for mood (Hürny et al, 1996a), the coarse indicator was less efficient than the multi-item reference scale for detection of chemotherapy side-effects, especially in situations with a low impact, such as completion of chemotherapy.

*Received 14 August 2000*
*Revised 19 January 2001*
*Accepted 9 February 2001*

*Correspondence to:* J Bernhard

It is less recognized that, in particular situations, single-item scales may be as efficient as multi-item scales. In the study cited above, the responsiveness of the indicator increased with the subjective impact of the clinical event and even exceeded that of the multi-item scale in case of disease recurrence (i.e., a major event) (Hürny et al, 1996a). The indicator was probably more influenced by factors other than mood related to the event, whereas the multi-item scale, assessing mood more precisely, was less subject to such influences. In other words, the impaired discriminant validity of the indicator was associated with an increased responsiveness. Discriminant validity of a measure refers to a higher correlation between this measure and the concepts intended to be measured than those not intended to be measured. Responsiveness to chemotherapy and course of disease are key criteria for clinical validity.

To further investigate the relationship between discriminant validity and responsiveness of these indicators, we compared them with standard measures of mental well-being and psychosocial functioning in patients with early breast cancer. Our hypothesis was that global single-item indicators are less precise for specific treatment effects (i.e., less discriminant validity) than multi-item scales but similarly efficient for overall treatment comparisons and changes over time because they reflect the summation of the individual meaning and importance of various factors for each patient.

### PATIENTS AND METHODS

### Sample

This study included a consecutive sample of patients with operable breast cancer from Sahlgrenska University Hospital in Göteborg which were randomized into one of the following IBCSG adjuvant therapy trials: Trial VI, for pre- and peri-menopausal, node-positive (N+) patients; Trial VII, for postmenopausal N+ patients; Trial VIII, for pre- and peri-menopausal, node-negative (N–) patients; Trial IX, for postmenopausal N-patients. In these trials, varying schedules of chemotherapy, endocrine therapy and their combinations were studied. The chemotherapy consisted of CMF (cyclophosphamide, methotrexate and 5-fluorouricil); the endocrine therapy was Tamoxifen or LH-RH (luteinizing hormone-releasing hormone) analogue. In patients with conservative surgery (quadrantectomy or lumpectomy) radiotherapy was started 2 weeks after the last chemotherapy course, or within 3 months in case of endocrine therapy alone. The randomization in Trials VI and VII was stratified by institution, type of surgery and oestrogen receptor (ER) status. The randomization in Trials VIII and IX was stratified by institution, ER status and radiotherapy.

Trials VI and VII started in July 1986 and were closed in April 1993 (Hürny et al, 1996b), (International Breast Cancer Study Group, 1996, 1997). Trial VIII started in March 1990 and was closed in October 1999. Trial IX started in October 1988 and was closed in August 1999. For this investigation, patients were enrolled between April 4, 1990, and November 27, 1992. Patient characteristics of the study sample were compared with those of all patients randomized into Trials VI to IX in Sweden between July 22, 1986 and November 24, 1993 (total Swedish sample).

### Data collection procedure

Patients were approached by a research nurse within 6 weeks after primary surgery and after being randomized into 1 of the 4 IBCSG trials but before starting adjuvant treatment. Besides the IBCSG QL form assessed in hospital, those patients who agreed to participate in this study were asked to fill in a set of additional questionnaires at home and to send it back to the local data manager. Both the IBCSG QL form and the additional questionnaires were assessed at baseline and at months 3 and 6 of adjuvant therapy. Clinical and sociodemographic data were part of the documentation of the IBCSG trials.

### Indicator and standard measures

4 LASA indicators were incorporated in the IBCSG QL form: physical well-being (PWB) (Priestman and Baum, 1976), mood (Priestman and Baum, 1976; Hürny et al, 1996a) and effort to cope (PACIS) (Hürny et al, 1993) were designed as global indicators, appetite as a more specific indicator for cytotoxic side-effects (Bernhard et al, 1997). All indicators were scored by measuring in millimetres from 0 to 100 and were reversed, with higher numbers reflecting better QL (e.g., less effort to cope). Concurrent validity (Butow et al, 1991), test–retest-reliability (Coates et al, 1990) and responsiveness to chemotherapy (Hürny et al 1992) have previously been documented. A 28-item adjective checklist for emotional well-being (Bf-S) (Zerseen, 1986) was also included in the IBCSG QL form. The Bf-S was transformed into scores from 0 to 100, with higher numbers reflecting better emotional well-being. In clinical trials, the global indicators were particularly relevant endpoints (Coates et al, 1987; Hürny et al, 1996b; Bernhard et al, 1999b). We capitalize on this experience.

To target the broad construct of psychosocial adaptation, we selected different domains. Mental well-being was measured by the Mood Adjective Check List (MACL) (Sjöberg et al, 1979). It contains 71 adjectives which are aggregated into 6 bipolar dimensions: pleasantness/unpleasantness, activation/deactivation, calmness/tension, extraversion/introversion, positive/negative social orientation and control/lack of control. Each dimension and an overall score (MACL TOT) is scored from 1 to 4, with higher numbers reflecting better mood. In various chronic conditions, the first 3 dimensions were of particular clinical relevance (Sullivan et al, 1993). We capitalize on this experience.

The Hospital Anxiety and Depression Scale (HAD) (Zigmond and Snaith, 1983) was used as a complement to the MACL. The HAD contains 14 items which are aggregated into summary scores for anxiety and depression ranging each from 0 to 21, with higher numbers reflecting more mood disturbance. The validated classification of psychiatric morbidity regarding non-psychiatric cases (scores 0–7), possible cases (scores 8–10) and probable cases (scores 11–21) was also tested for the Swedish version in patients with chronic disease or injury (Sullivan et al, 1993). We used a dichotomization with the cut-off score of ≥8.

Emotional behaviour (EB) and social interaction (SI), the main psychosocial dimensions of the Sickness Impact Profile (SIP) (Bergner et al, 1981), were chosen to assess health-related dysfunction in personal and social life (Ahlmén et al, 1990; Sullivan et al, 1993). The SIP/EB contains 9 statements indicative of depression, anxiety, low self-esteem and lack of control, the SIP/SI includes 20 statements on quality and quantity of social interaction within and outside the family. For each dimension, the percentage of maximum dysfunction is calculated according to predetermined weights, ranging from 0 to 100 (most dysfunction). Based on experiences in Sweden (Sullivan et al, 1986), limits for no (score = 0), slight to moderate (scores 1–10) and marked dysfunction (scores 11–100) were defined (Augustinsson et al, 1989). We used a dichotomization with the cut-off score of ≥11.

### Statistical methods

Submission rates of the IBCSG QL form including the indicator measures and of the questionnaires including the comparison measures were calculated as the ratio of numbers of received versus expected questionnaires of all patients randomized in the participating hospital during the study period separately for each time point.

Convergent and discriminant validity of the indicators were investigated by a multitrait-multimethod correlation analysis (Ahlmén et al, 1990; Sullivan et al, 1993), created from hypotheses about measures targeting the same (convergent) versus different concepts (discriminant validity). A matrix was developed for baseline and month 6. Correlation coefficients were considered low (<0.4), moderate-to-high (0.4–0.7) and substantial (>0.7) (Ware et al, 1993).

Responsiveness to chemotherapy and changes over time were tested by standardized response mean (SRM; mean change/SD of this change) (Liang et al, 1990; Katz et al, 1992). Randomized treatment assignments were grouped across the whole study sample separately for the first 3 and 6 months on study as shown in Table 1. Chemo-endocrine therapy was grouped together with

© 2001 Cancer Research Campaign

**Table 1** Biomedical and sociodemographic characteristics of the study sample and the total Swedish sample

| Characteristics | Study sample (*n* = 84) | | Total Swedish sample (*n* = 611) | |
|---|---|---|---|---|
| | *n* | % | *n* | % |
| Menopausal status | | | | |
| Premenopausal | 39 | 46 | 281 | 46 |
| Postmenopausal | 45 | 54 | 330 | 54 |
| Nodal status | | | | |
| Positive | 59 | 70 | 505 | 83 |
| Negative | 25 | 30 | 106 | 17 |
| Receptor status[a] | | | | |
| PR, negative | 29 | 34 | 231 | 38 |
| PR, positive | 55 | 66 | 370 | 62 |
| ER, negative | 10 | 12 | 133 | 22 |
| ER, positive | 74 | 88 | 472 | 78 |
| Type of surgery | | | | |
| Total mastectomy | 47 | 56 | 428 | 70 |
| Conservative surgery | 37 | 44 | 181 | 30 |
| Adjuvant treatment grouping[b] | | | | |
| Months 1–3 | | | n.a. | |
| Chemotherapy | 42 | 55 | | |
| Endocrine therapy | 21 | 28 | | |
| Chemo and endocrine therapy | 13 | 17 | | |
| Months 1–6 | | | n.a. | |
| Chemotherapy 3 months | 33 | 45 | | |
| Chemotherapy 6 months | 19 | 26 | | |
| Endocrine therapy only | 21 | 29 | | |
| Partnership | | | | |
| Yes | 57 | 68 | 449 | 74 |
| No | 27 | 32 | 159 | 26 |
| Employment | | | | |
| Yes | 53 | 63 | 399 | 65 |
| No | 31 | 37 | 210 | 35 |
| Level of education | | | | |
| Mandatory | 39 | 46 | 316 | 52 |
| Higher level | 45 | 54 | 292 | 48 |

[a] PR: progesterone receptor; ER: estrogen receptor. [b] Combined groups of randomized treatment groups are applicable to the study sample only.

chemotherapy. The SRMs were interpreted as trivial (<0.2), small (0.2–0.5), moderate (0.5–0.8) or large (>0.8) effect size (Cohen, 1977). We used Cohen's criteria as an illustrative measure and compared its threshold for a small effect to a minimal clinically significant change as defined in an adjuvant breast cancer trial using the same indicators (Hürny et al, 1996b).

We expected a substantial improvement in QL in patients with endocrine therapy only reflecting adaptation to disease, to a lesser extent in those with chemotherapy for the first 3 months reflecting treatment burden and no improvement in case of chemotherapy for the first 6 months (Hürny et al, 1996a; 1996b; Bernhard et al, 1997). Among the indicators, adaptation was expected to be most expressed in coping (PACIS) scores, chemotherapy sides-effects in coping and physical well-being. The 6-months grouping was selected as primary comparison and tested across all measures by the Friedman test. This test is related only to the pattern and therefore robust against variation of the single measurements. A sample size of $n = 70$ was considered as sufficient.

As a further issue of clinical validity, we explored whether the indicators are sensitive to subgroups of patients according to their levels of mental well-being and psychosocial functioning. The HAD and SIP scores were chosen as criterion measures. Known-groups comparisons of the dichotomized absolute scores were used at baseline and at month 6. Lines indicating 95% CI around observed mean effects were chosen to show the consistency of patterns.

### RESULTS

### Sample description and baseline scores

During the study period, 101 patients were randomized into IBCSG Trials VI to IX at the Sahlgrenska University Hospital and were asked to participate in this additional investigation. 88 of these patients (87%) agreed but 4 were ineligible for the IBCSG trials. In the remaining sample ($n = 84$), the submission rate of both the IBCSG QL form including the indicators and the set including the comparison measures was 96% at baseline, 90% at month 3 and 89% at month 6. At each timepoint, the sample size was varying by QL measure due to missing data on available questionnaires (LASA indicators: 0–3%, Bf-S: 4–13%, comparison measures: 0–2% by measure and timepoint).

Biomedical and sociodemographic characteristics of the study sample and of the total Swedish sample are summarized in Table

 *© 2001 Cancer Research Campaign*

**Table 2** Mean values of the QL indicators in the study sample and the total Swedish sample at baseline by menopausal status

| QL indicators[a] | Menopausal status | Study sample *n* | Study sample Mean (CI, 95%) | Total Swedish sample *n* | Total Swedish sample Mean (CI, 95%) |
|---|---|---|---|---|---|
| Physical well-being | pre | 37 | 70.0 (61.6–78.5) | 248 | 71.0 (68.1–74.0) |
| | post | 41 | 65.8 (57.5–74.1) | 255 | 69.3 (66.3–72.3) |
| Appetite | pre | 37 | 76.2 (67.1–85.4) | 248 | 79.3 (76.4–82.2) |
| | post | 41 | 78.4 (71.3–85.6) | 256 | 78.3 (75.3–81.2) |
| Mood | pre | 37 | 57.4 (48.6–66.3) | 248 | 59.7 (56.6–62.8) |
| | post | 41 | 64.0 (56.1–71.9) | 255 | 62.1 (59.1–65.0) |
| PACIS | pre | 37 | 58.6 (49.0–68.2) | 245 | 56.1 (52.8–59.5) |
| | post | 40 | 59.2 (49.7–68.6) | 252 | 61.1 (58.0–64.2) |
| Bf-S | pre | 36 | 69.5 (61.2–77.9) | 236 | 68.2(65.2–71.3) |
| | post | 39 | 72.3 (65.0–79.5) | 244 | 71.7 (68.9–74.5) |

[a]All scales range from 0 to 100, with higher scores indicating better QL.

1. The study sample included 84 patients, with a mean age of 56 years (range: 31–75 years); the total sample included 611 patients of the same age (mean = 56 years, range: 28–79 years). The study sample had a higher rate of patients with conservative surgery, N– and ER+ status. A similar and unremarkable sociodemographic situation of both samples was noted compared with official statistics of Sweden, except a lower percentage of employment. Overall, the study sample was comparable to the total sample.

A majority of patients in the study sample underwent chemotherapy during the first 6 months on study. 11% of all patients started radiotherapy before month 3, and 21% between months 3 and 6. No case of disease recurrence was registered within the first 6 months.

The baseline scores of the indicators are shown in Table 2. The 2 samples showed comparable scores. A tendency toward higher scores (i.e., better QL) was present in all indicators. Overall, the mood and coping scores were most impaired.

### Convergent and discriminant validity

The multitrait-multimethod matrix is shown for the scores at baseline and month 6 in Table 3. Measures targeting the same (convergent) versus different concepts (discriminant validity) were investigated both within the indicators and standard measures and among all measures. It has to be noted that convergent measures included in the same questionnaire are generally expected to be more highly correlated than those of separate questionnaires.

Regarding the indicators at baseline, the 2 measures of emotional well-being (mood, Bf-S) showed the highest correlation (r = 0.72). The PACIS was moderately related to both physical and emotional measures (0.42–0.62), thus referring to a separate construct. Among the standard measures, emotional scales of different instruments (MACL subscales, HAD anxiety and depression, SIP emotional behaviour) were closer correlated with each other (0.38–0.76) than with SIP social interaction (0.29–0.53).

Taking into account both indicator and standard measures at baseline, the mood indicator was most strongly correlated with the MACL pleasantness (r = 0.77) and total score (r = 0.71) and with HAD depression (r = 0.69). The complementary adjective checklist Bf-S reflected the same pattern with more substantial correlations. The indicators for coping (0.53–0.65) and physical well-being (0.42–0.62) showed lower correlations with the mental well-being measures than the mood indicator (0.61–0.77), and they were more highly correlated with mental well-being and emotional functioning than with social functioning; SIP social interaction was only marginally associated.

The matrix at month 6 is based on scores from patients with different adjuvant treatments. The correlation coefficients were not adjusted for treatment group to investigate discriminant validity under treatment overall. Among the indicators, mood and Bf-S were both strongly correlated with physical well-being (r = 0.80 and 0.78, respectively). Coping was again moderately correlated with both physical and emotional measures (0.48–0.62). Among the standard measures, emotional scales of different instruments (MACL subscales, HAD anxiety and depression, SIP emotional behaviour) were again closer correlated with each other (0.48–0.84) than with SIP social interaction (0.43–0.66). The coefficients between corresponding indicator and standard measures were generally lower than at baseline. The mood indicator was again most but only moderately correlated with the MACL pleasantness (r = 0.61) and total score (r = 0.60) and showed the same pattern of relationships as the Bf-S. Coping was again more highly correlated with mental well-being and emotional functioning (0.52–0.63) than it was with SIP social interaction (r = 0.45). Physical well-being showed a similar pattern, despite the high correlation with mood.

In summary, the correlation analyses at baseline showed convergent and discriminant patterns among the indicator and standard measures in accordance with their construct. In contrast to the standard measures, the patterns among the indicators showed less discriminant validity under treatment than at baseline.

### Responsiveness to chemotherapy and changes over time

Responsiveness of the indicators and the mental well-being measures to chemotherapy and changes over time were evaluated over the first 3 and 6 months from randomization. The mood indicator provided reference data for a minimal clinically significant change. In IBCSG Trial VII, postmenopausal patients who did not

© 2001 Cancer Research Campaign 

**Table 3** Multitrait-multimethod matrix of indicator and standard measures at baseline (*n*: 78–88) and at month 6 (*n*: 65–77)[a]

| | Indicator measures | | | | | Standard comparison measures | | | | | | | |
|---|---|---|---|---|---|---|---|---|---|---|---|---|---|
| | Physical well-being | | Emotional well being | | Adjustment | Mental well-being | | | | | | Psychosocial functioning | |
| | **PWB** | **Appetite** | **Mood** | **Bf-SH[b]** | **PACIS** | **MACL pleas** | **MACL act** | **MACL calm** | **MACL TOT** | **HAD anxiety** | **HAD depress** | **SIP Emot** | **SIP social** |
| PWB | | 0.44 | 0.68 | 0.54 | 0.58 | 0.50 | 0.62 | 0.42 | 0.51 | –0.46 | –0.59 | –0.47 | –0.29 |
| Appetite | 0.62 | | 0.53 | 0.49 | 0.42 | 0.49 | 0.56 | 0.38 | 0.54 | –0.39 | –0.56 | –0.23 | –0.32 |
| Mood | **0.80** | 0.52 | | **0.72** | 0.62 | **0.77** | 0.69 | 0.61 | **0.71** | –0.63 | –0.69 | –0.47 | –0.29 |
| Bf–S | **0.78** | 0.63 | 0.63 | | 0.59 | **0.75** | 0.67 | **0.70** | **0.76** | **–0.70** | **–0.72** | –0.61 | –0.50 |
| PACIS | 0.48 | 0.48 | 0.49 | 0.62 | | 0.65 | 0.55 | 0.53 | 0.58 | –0.60 | –0.57 | –0.39 | –0.25 |
| MACL Pleas | 0.57 | 0.47 | 0.61 | **0.74** | 0.62 | | **0.78** | **0.84** | **0.94** | **–0.74** | **–0.73** | –0.48 | –0.33 |
| MACL Act | 0.65 | 0.60 | 0.55 | **0.70** | 0.52 | **0.75** | | 0.69 | **0.86** | –0.56 | **–0.76** | –0.38 | –0.29 |
| MACL Calm | 0.54 | 0.50 | 0.55 | 0.69 | 0.53 | **0.85** | **0.73** | | **0.90** | **–0.75** | –0.66 | –0.53 | –0.40 |
| MACL TOT | 0.62 | 0.52 | 0.60 | **0.77** | 0.56 | **0.93** | **0.88** | **0.90** | | **–0.71** | **–0.74** | –0.46 | –0.39 |
| HAD Anxiety | –0.47 | –0.55 | –0.46 | –0.69 | –0.63 | **–0.81** | –0.65 | **–0.84** | **–0.80** | | **0.73** | **0.73** | 0.40 |
| HAD Depress | –0.45 | –0.51 | –0.51 | –0.61 | –0.54 | **–0.71** | –0.67 | –0.65 | **–0.74** | **0.78** | | 0.56 | 0.34 |
| SIP Emot | –0.42 | –0.51 | –0.37 | –0.57 | –0.57 | –0.62 | –0.48 | –0.57 | –0.59 | 0.66 | 0.63 | | 0.53 |
| SIP Social | –0.39 | –0.44 | –0.34 | –0.54 | –0.45 | –0.49 | –0.46 | –0.43 | –0.50 | 0.48 | 0.56 | 0.66 | |

[a]The Pearson correlation coefficients above the diagonal represent the scores at baseline, those below the scores at month 6. Correlation coefficients are considered low (<0.4), moderate-to-high (0.4–0.7) and substantial (>0.7: in bold). Varying sample size is due to partial missing data. [b]The Bf-S is a 28-item scale included in the form of the indicators as internal reference measure.

*© 2001 Cancer Research Campaign*

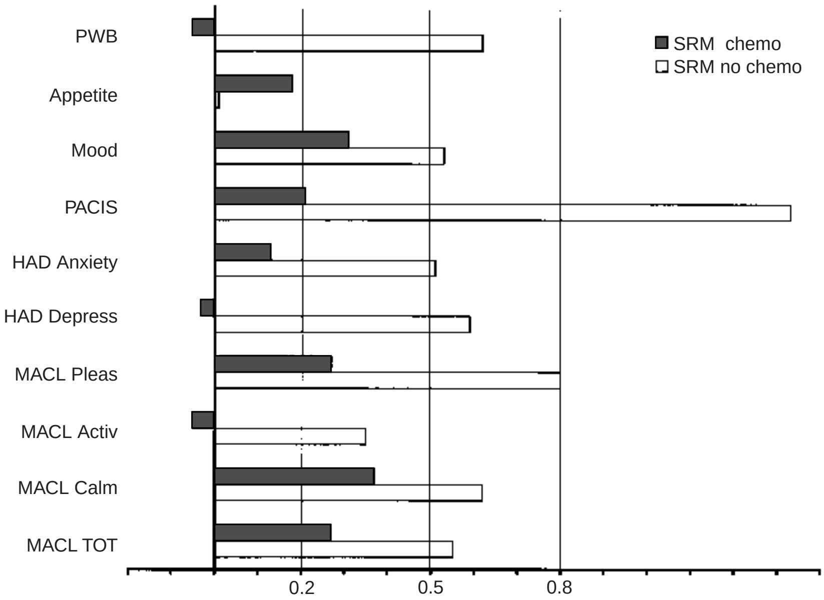

**Figure 1** Standardized response means (SRM) for indicator and standard comparison measures by treatment group between baseline and month 3. SRM are considered trivial (<0.2), small (0.2–0.5), moderate (0.5–0.8) and large (>0.8). Positive values indicate an improvement

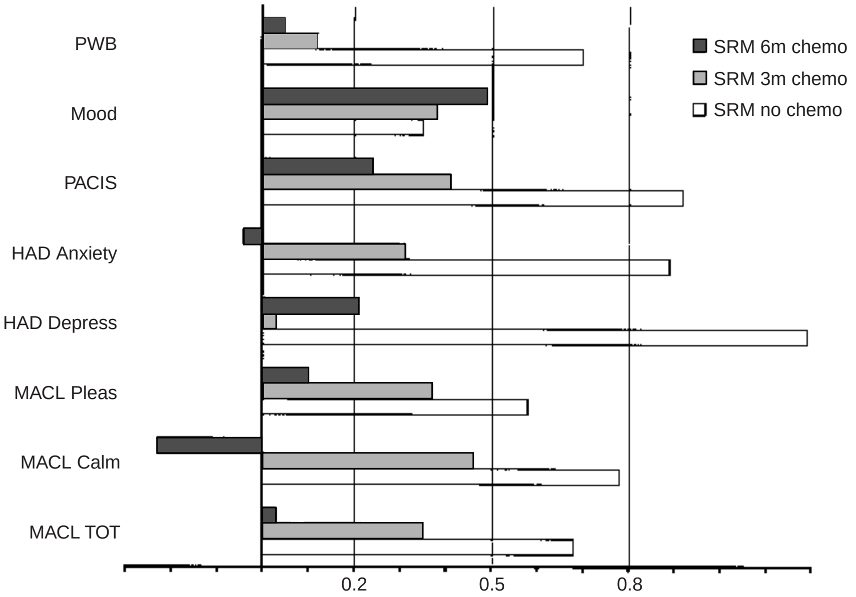

**Figure 2** Standardized response means (SRM) for selected indicator and standard comparison measures by treatment group between baseline and month 6. SRM are considered trivial (<0.2), small (0.2–0.5), moderate (0.5–0.8) and large (>0.8). Positive values indicate an improvement receive prior chemotherapy indicated an average within-patient deterioration of 3.6% of full scale range (i.e., 0–100; $P = 0.05$) at the beginning of delayed chemotherapy (Hürny et al, 1996b). This effect corresponds to a SRM of 0.14 in the group without early chemotherapy and to 0.18 in that with chemotherapy for 6 months. It is close to the threshold value of 0.2 for a small effect (Figures 1 and 2). The group with chemotherapy included different treatment schedules and the number of patients in each group was small. Therefore, only the main effects are to be interpreted.

For the 3-months comparison, the 4 indicators were compared with the HAD anxiety and depression scales, the MACL pleasantness, activity and calmness scales and the MACL total score. This comparison included patients receiving chemotherapy (with or without endocrine therapy) versus endocrine therapy only for the first 3 months.

Figure 1 shows the SRMs separately for each measure and treatment group between baseline and month 3. In patients with no early chemotherapy, the SRM indicated the expected improvement in all measures of at least moderate degree, with the exceptions of appetite (0.01) and MACL activity (0.35). Large effects were present for coping (1.33) and MACL pleasantness (0.80). In patients with chemotherapy in this period, the SRM indicated a small improvement in mood (0.31), coping (0.21), MACL pleasantness (0.27), calmness (0.37) and total score (0.27). Physical well-being did not change and differed from emotional measures, in agreement with the MACL activity

© 2001 Cancer Research Campaign scale. The coping indicator was most responsive to the presence or absence of chemotherapy, followed by physical well-being and the HAD depression scale.

For the 6-months comparison, we selected those measures with an SRM of at least moderate degree in either group of the 3-months comparison. This comparison included patients receiving chemotherapy (with or without endocrine therapy) for 6 months versus chemotherapy (with or without endocrine therapy) for the first 3 months versus endocrine therapy only for the first 6 months.

Figure 2 shows the SRMs separately for the selected measures and the 3 treatment groups between baseline and month 6. In patients with no early chemotherapy, the SRM showed the expected improvement of moderate to large degree in all measures, with the exception of mood (SRM = 0.35). In patients with chemotherapy for the first 3 months, the SRM indicated a similar pattern of a small improvement as in the 3 month period, with comparable responsiveness of the indicators for mood (0.38) and coping (0.41), the MACL pleasantness (0.37), calmness (0.46) and total score (0.35), and HAD anxiety (0.31). In patients with chemotherapy for 6 months, the SRM were again smaller, with the exception of mood (0.49), indicating no or only small changes. The only and small deterioration relative to baseline was noted for MACL calmness (–0.23). The latter scale was most responsive to the distinction between 3 and 6 months of chemotherapy, followed by MACL pleasantness and HAD anxiety. The predicted dose–response pattern was present both for the standard measures (HAD anxiety, MACL) and the indicators (physical well-being, coping) ($P$ = 0.008).

In summary, the indicators reflected the presence or absence of early chemotherapy at least as well as the standard measures but were less sensitive to the duration of chemotherapy.

### Distinguishing groups by levels of mental distress and psychosocial dysfunction

The indicators' responsiveness to clinically validated levels of mental distress and psychosocial dysfunction (i.e., 'case' versus 'non-case') was investigated for scores at baseline and month 6. The evaluation at month 6 was based on absolute scores without adjustment for baseline or treatment.

Figure 3 shows the indicator scores according to HAD anxiety (non-cases $n$ = 54; cases: $n$ = 27) and depression (non-cases $n$ = 74; cases: $n$ = 7) at baseline. All of the indicators reflected the presence or absence of a possible or probable mood disorder in the expected direction. The only marked overlap of confidence intervals regarding non-cases and cases was in the prediction of physical well-being by depression, the number of cases being at the lower limit for this type of illustration. The distinction was most pronounced for mood and coping. Patients with anxiety beyond the cut-off level reported a similar level of mood (mean = 43) and coping (mean = 37) which was remarkably lower compared to that of the non-cases (mood: mean = 71; coping: mean = 68). Depression yielded similar figures, with low levels in cases (mood: mean = 28; coping: mean = 30) and substantially higher levels in non-cases (mood: mean = 65; coping: mean = 61).

Figure 4 shows the indicator scores according to SIP emotional behaviour (non-cases = no or slight-to-moderate dysfunction: $n$ = 56; cases = marked dysfunction: $n$ = 25) and social interaction (non-cases: $n$ = 67; cases: $n$ = 14) at baseline. Regarding emotional behaviour, all of the indicators reflected the presence or absence of dysfunction in the expected direction. Mood and coping were again most sensitive. Cases with marked emotional dysfunction reported a low level of mood (mean = 47) and coping (mean = 42)

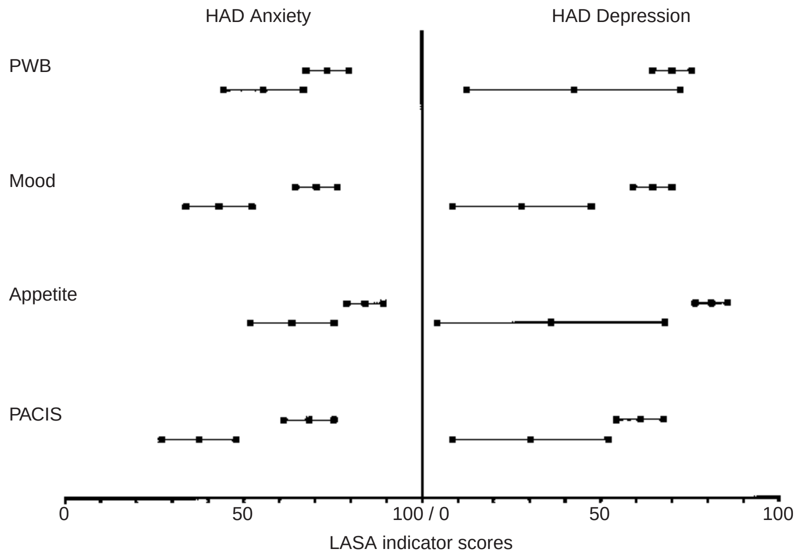

**Figure 3** Mean scores with 95% Cl of the indicator measures by HAD anxiety and depression at baseline. For each indicator, scores of non-psychiatric cases according to the HAD criterion measure are shown as first line, scores of possible and probable cases as second line. Higher scores of the indicators refer to better QL

*© 2001 Cancer Research Campaign*

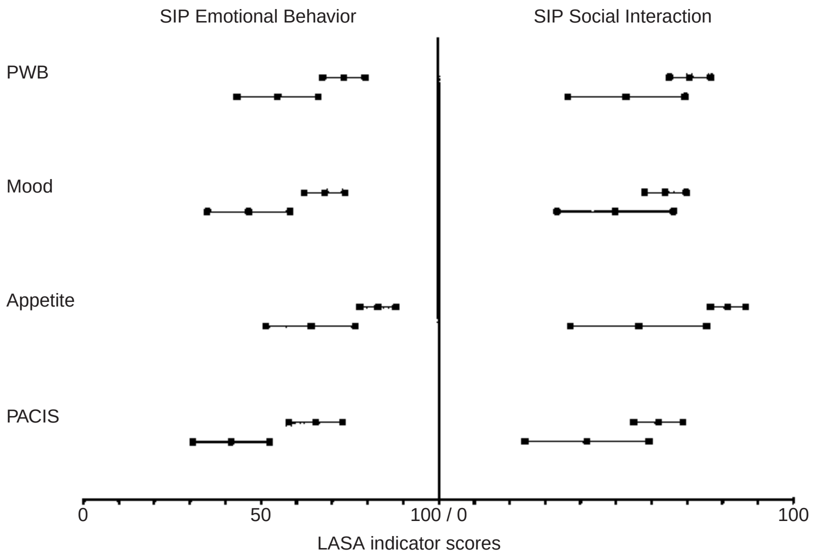

**Figure 4** Mean scores with 95% CI of the indicator measures by SIP emotional behaviour and social interaction at baseline. For each indicator, scores of no more than moderate dysfunction according to the SIP criterion measure are shown as first line, scores of marked dysfunction as second line. Higher scores of the indicators refer to better QL

which clearly contrasted to that of the non-cases (mood: mean = 68; coping: mean = 65). There were similar findings regarding social interaction (SIP), with some overlap of confidence intervals in physical well-being, mood and coping probably due to the small number of cases.

At month 6, the findings were consistent with those at baseline. All of the indicators reflected the absence ($n$ = 54) or presence ($n$ = 21) of anxiety (HAD) and the absence ($n$ = 58) or presence ($n$ = 17) of marked emotional dysfunction (SIP), with the largest differences again for mood and coping. There was some overlap of confidence intervals in all indicators regarding non-cases ($n$ = 68) and cases ($n$ = 7) of depression (HAD) and non-cases ($n$ = 63) and cases ($n$ = 12) of marked social dysfunction (data not shown).

### DISCUSSION

In a consecutive Swedish sample of patients with early breast cancer, we investigated the hypothesis that global QL indicators assessed with single items have less discriminant validity and thus are less precise for specific treatment effects than multi-item scales but similarly efficient for overall treatment comparisons and changes over time.

The standard measures indicated the expected impairment in this situation (Fallowfield et al, 1990; Maunsell et al, 1996), characterized by anxiety rather than depression (Maraste et al, 1992). At baseline, the indicators and standard measures showed convergent and discriminant patterns in accordance with their concept. For example, the mood indicator yielded the same pattern with the standard measures as the adjective checklist for emotional well-being (Bf-S) but showed lower correlations in consequence of its lower reliability.

Under treatment, these patterns were less convergent or discriminant as compared to baseline. In particular, mood and physical well-being were substantially correlated. Obviously, the indicator and standard measures were affected differently by the various treatment regimens. To get an overall impression, we did not adjust this analysis for treatment. The question is how the lower discriminant validity of the indicators does affect their responsiveness to chemotherapy and changes over time.

In patients without chemotherapy, both the global indicators and the standard measures reflected the adaptation to the disease. Among the indicators this change was most obviously expressed in perceived coping effort, the most subjective measure (Hürny et al, 1993). This finding speaks for a summative effect of various factors.

In patients with 3 months chemotherapy, the responsiveness was comparable between the indicators for mood and coping and the MACL pleasantness and calmness scales, whereas the HAD depression scores were almost stable. As a reflection of treatment burden, patients receiving chemotherapy for 6 months showed no improvement. An exception was mood. This indicator and that for physical well-being showed clearly different patterns despite the unusually high proportion of variance (64%) explained by each other.

Overall, the standard measures reflected the distinction between 3 and 6 months of chemotherapy better than the indicators. However, in regard to the more sharply contrasting situations of patients with and without chemotherapy, the indicators showed at least comparable performance. In other words, their lower discriminant validity did not result in less responsiveness to 2 markedly different clinical situations.

From a psychometric point of view, the lower precision of the indicators under treatment questions their validity as outcome measures. This may be less so from a clinical point of view. It is common sense that patients' perception of disease and treatment burden is more of a global nature than subdivided into highly specific domains as assessed by the standard measures. The contributing clinical factors may substantially change over time. The pattern of response among the indicators speaks again for a summative effect reflecting the individual meaning and importance of various factors.

This property may also explain the responsiveness of the indicators to mental distress and psychosocial dysfunction. All were

*© 2001 Cancer Research Campaign*

sensitive to these conditions both at baseline and under treatment. In accordance with their concept, the indicators for mood and coping were most sensitive. Adjustment to breast cancer is known to be associated with mental distress and psychiatric morbidity (Watson et al, 1991), irrespective of any causal interaction. Physical well-being and appetite also reflected the criterion measures well. Patients under higher psychological distress are expected to report more physical symptoms (Watson and Pennebaker, 1989), as shown in the situation of adjuvant therapy for breast cancer (Manne et al, 1994).

Serious psychosocial impairment is only partly determined by cytotoxic side-effects but influenced by multiple individual factors such as a history of depression (Maunsell et al, 1992). In case of relatively mild regimens, as in this study, there is evidence that adaptation to the disease is more important for patient's QL than cytotoxic side-effects (Hürny et al, 1996b). Identifying patients with poor adaptation is relevant for subgroup analyses, for example in developing risk-adapted treatment strategies or supportive interventions. Given the large sample sizes of phase-III trials, these indicators may carry this type of information sufficiently well. However, the sensitivity of the HAD as screening instrument has recently been questioned, especially regarding depression (Hall et al, 1999). Our findings have to be interpreted within these limitations.

The evaluation of single-item measures has frequently been restricted to cross-sectional comparison with standard measures (McCormack et al, 1988; Cunny and Perri, 1991). McHorney et al investigated how precisely different methods for measuring general health status discriminated between different groups of patients (McHorney et al, 1992). Their results suggested that roughly twice the sample size would be required for a single-item measure to achieve the precision of a long-form (multi-item) measure. A cancer clinical trial is a different situation. Given that disease and treatment factors may change considerably only a longitudinal comparison gives sufficient information to judge the properties of QL measures.

The rather small sample limits the generalization of our findings, although it was sufficiently large to demonstrate the expected pattern, in agreement with previous studies in early (Hürny et al, 1996b) and advanced disease (Coates et al, 1987). The sensitivity to performance status (Bernhard et al, 1999a), disease recurrence (Hürny et al, 1996b) and tumour response (Bernhard et al, 1999b), and their prognostic value for survival (Coates et al, 1992, 1993;) add evidence to the clinical relevance of these indicators.

In conclusion, LASA indicators were confirmed to be responsive to cytotoxic side effects, mental distress and psychosocial dysfunction in patients with early breast cancer. According to our hypothesis, the lower reliability of single as opposed to multi-item scales affects more their discriminant validity than responsiveness. This is less decisive for treatment comparisons in large sample sizes.

### ACKNOWLEDGEMENTS

We thank patients, physicians, nurses, and data managers who participated in the International Breast Cancer Study Group (IBCSG) trials. We gratefully acknowledge the support for this project provided by the Swedish Cancer Fund and the Medical Faculty, Göteborg University. We are especially grateful to Anita Larsson for local data management and to Nils-Gunnar Pehrsson for statistical analysis and advice. We acknowledge the initial support of IBCSG trials provided by the Ludwig Institute for Cancer Research, the Cancer League of Ticino, and the Swiss Cancer League. We further acknowledge the continuing support for central coordination, data management, and statistics provided by the Swedish Cancer League, the Australian Cancer Society, the National Health and Medical Research Council of Australia (grant numbers 890028, 910420, 920876, 950328, 980379), the Australian New Zealand Breast Cancer Trials Group, the Frontier Science and Technology Research Foundation, the Swiss Group for Clinical Cancer Research, the United States National Cancer Institute (CA-75362), and the American Cancer Society (grant RPG-90-013-08-PBP).

### REFERENCES

Ahlmén EM, Bengtsson CB, Sullivan BM and Bjelle A (1990) A comparison of overall health between patients with rheumatoid arthritis and a population with and without rheumatoid arthritis. *Scand J Rheumatol* **19**: 413–421

Augustinsson LE, Sullivan L and Sullivan M (1989) Chronic pain in functional neurosurgery: Function and mood in various diagnostic groups with reference to epidural spinal electrical stimulation. *Schmerz/Pain/Douleur* **10**: 30–40

Bergner M, Bobbit RA, Carter WB and Gilson BS (1981) The Sickness Impact Profile: development and final revision of a health status measure. *Med Care* **19**: 787–805

Bernhard J, Hürny C, Coates AS, Peterson HF, Castiglione Gertsch M, Gelber RD, Goldhirsch A, Senn HJ and Rudenstam CM (1997) Quality of life assessment in patients receiving adjuvant therapy for breast cancer: the IBCSG approach. The International Breast Cancer Study Group. *Ann Oncol* **8**: 825–835

Bernhard J, Castiglione Gertsch M, Schmitz SF, Thürlimann B, Cavalli F, Morant R, Fey MF, Bonnefoi H, Goldhirsch A and Hürny C (1999a). Quality of life in postmenopausal patients with breast cancer after failure of tamoxifen: formestane versus megestrol acetate as second-line hormonal treatment. Swiss Group for Clinical Cancer Research (Sakk). *Eur J Cancer* **35**: 913–920

Bernhard J, Thürlimann B, Schmitz SF, Castiglione Gertsch M, Cavalli F, Morant R, Fey MF, Bonnefoi H, Goldhirsch A and Hürny C (1999b) Defining clinical benefit in postmenopausal patients with breast cancer under second-line endocrine treatment: does quality of life matter? *J Clin Oncol* **17**: 1672–1679

Butow P, Coates A, Dunn S, Bernhard J and Hürny C (1991) On the receiving end IV: Validation of quality of life indicators. *Ann Oncol* **2**: 597–603

Coates A, Gebski V, Bishop JF, Jeal PN, Woods RL, Snyder R et al and for the Australian-New Zealand Breast Cancer Trials Group (1987) Improving the quality of life during chemotherapy for advanced breast cancer. A comparison of intermittent and continuous treatment strategies. *N Engl J Med* **317**: 1490–1495

Coates A, Glasziou P and McNeil D (1990) On the receiving end – III. Measurement of quality of life during cancer chemotherapy. *Ann Oncol* **1**: 213–217

Coates A, Gebski V, Signorini D, Murray P, McNeil D and Byrne M et al and for the Australian New Zealand Breast Cancer Trials Group (1992) Prognostic value of quality-of-life scores during chemotherapy for advanced breast cancer. *J Clin Oncol* **10**: 1833–1838

Coates A, Thomson D, McLeod GR, Hersey P, Gill PG, Olver IN, Kefford R, Lowenthal RM Beadle G and Walpole E (1993) Prognostic value of quality of life scores in a trial of chemotherapy with or without interferon in patients with metastatic malignant melanoma. *Eur J Cancer* **29A**: 1731–1734

Cohen J (1977) *Statistical power analysis for the behavioral sciences*. Academic Press: New York

Cunny KA and Perri M (1991) Single-item vs multiple-item measures of health-related quality of life. *Psychological Reports* **69**: 127–130

Fallowfield LJ, Hall A, Maguire GP and MB (1990) Psychological outcomes of different treatment policies in women with early breast cancer outside a clinical trial. *Br Med J* **301**: 575–580

Hall A, A'Hern R and Fallowfield L (1999) Are we using appropriate self-report questionnaires for detecting anxiety and depression in women with early breast cancer? *Eur J Cancer* **35**: 79–85

Hürny C, Bernhard J, Gelber RD, Coates A, Castiglione M, Isley M, Dreher D, Peterson H, Goldhirsch A and Senn HJ (1992) Quality of life measures for patients receiving adjuvant therapy for breast cancer: an international trial. The International Breast Cancer Study Group. *Eur J Cancer* **28**: 118–124

Hürny C, Bernhard J, Bacchi M, van Wegberg B, Tomamichel M, Spek U, Coates A, Castiglione M, Goldhirsch A and Senn HJ et al (1993) The Perceived Adjustment to Chronic Illness Scale (PACIS): a global indicator of coping for

© 2001 Cancer Research Campaign operable breast cancer patients in clinical trials. Swiss Group for Clinical Cancer Research (SAKK) and the International Breast Cancer Study Group (IBCSG) *Support Care Cancer* **1**: 200–208

Hürny C, Bernhard J, Coates A, Peterson HF, Castiglione-Gertsch M, Gelber RD, Rudenstam CM, Collins J, Lindtner J, Goldhirsch A and Senn HJ (1996a) Responsiveness of a single-item indicator versus a multi-item scale: assessment of emotional well-being in an international adjuvant breast cancer trial. *Med Care* **34**: 234–248

Hürny C, Bernhard J, Coates AS, Castiglione Gertsch M, Peterson HF, Gelber RD, Forbes JF, Rudenstam CM, Simoncini E, Crivellari D, Goldhirsch A and Senn HJ (1996b) Impact of adjuvant therapy on quality of life in women with node-positive operable breast cancer. International Breast Cancer Study Group. *Lancet* **347**: 1279–1284

Katz JN, Larson MG, Phillips CB, Fossel AH and Liang MH (1992) Comparative measurement sensitivity of short and longer health status instruments. *Med Care* **30**: 917–925

Liang MH, Fossel AH and Larson MG (1990) Comparisons of five health status instruments for orthopedic evaluation. *Med Care* **28**: 632–642

Manne SL, Sabbioni M, Bovbjerg DH, Jacobsen PB, Taylor KL and Redd WH (1994) Coping with chemotherapy for breast cancer. *J Behav Med* **17**: 41–55

Maraste R, Brandt L, Olsson H and Ryde Brandt B (1992) Anxiety and depression in breast cancer patients at start of adjuvant radiotherapy. Relations to age and type of surgery. *Acta Oncol* **31**: 641–643

Maunsell E, Brisson J and Deschênes L (1992) Psychological distress after initial treatment of breast cancer. *Cancer* **70**: 120–125

Maunsell E, Brisson J, Deschenes L and Frasure Smith N (1996) Randomized trial of a psychologic distress screening program after breast cancer: effects on quality of life. *J Clin Oncol* **14**: 2747–2755

McCormack HM de L. Horne DJ and Sheather S (1988) Clinical applications of visual analogue scales: a critical review. *Psych Med* **18**: 1007–1019

McHorney CA, Ware JE, Rogers WH and et al (1992) The validity and relative precision of MOS Short- and Long-Form Health Status Scales and Dartmouth COOP charts: Results from the medical outcomes study. *Med Care* **30**(suppl): MS253–MS265

Priestman TJ and Baum M (1976) Evaluation of quality of life in patients receiving treatment for advanced breast cancer. *Lancet* **i**: 899–900

Sjöberg L, Svensson E and Persson LO (1979) The measurement of mood. *Scand J Psychol* **20**: 1–18

Sullivan BM, Ahlmén M, Archenholtz B and Svensson G (1986) Measuring health in rheumatic disorders by means of a Swedish version of the sickness impact profile. *Scand J Rheumatol* **15**: 193–200

Sullivan M, Karlsson J, Sjöström L, Backman L, Bengtsson C, Bouchard C, Dahlgren S, Jonsson E, Larsson B, Lindstedt S, Näslund I, Olbe L and Wedel H (1993) Swedish obese subjects (SOS) – an intervention study of obesity. Baseline evaluation of health and psychosocial functioning in the first 1743 subjects examined. *Int J Obes* **17**: 503–512

Ware JE, Snow KK, Kosinski M and Gandek B (1993) *SF-36 Health Survey. Manual and interpretation guide*. The Health Institute, New England Medical Center: Boston

Watson D and Pennebaker JW (1989) Health complaints, stress, and distress: Exploring the central role of negative affectivity. *Psychol Rev* **96**: 234–254

Watson M, Greer S, Rowden L, Gorman C, Robertson B, Bliss JM and Tunmore R (1991) Relationships between emotional control, adjustment to cancer and depression and anxiety in breast cancer patients. *Psychol Med* **21**: 51–57

Zerssen Dv (1986) Clinical self-rating scales (CSRS) of the Munich Psychiatric Information System (PSYCHIS München). In: *Assessment of depression* Sartorius N and Ban TA (eds) pp. 270–303. Springer: Berlin

Zigmond AS and Snaith RP (1983) The Hospital Anxiety and Depression Scale. *Acta Psychiatrica Scand* **67**: 362–370

*© 2001 Cancer Research Campaign* 
